# Genetic Variants in the Promoter Region of* miR-10b* and the Risk of Breast Cancer

**DOI:** 10.1155/2017/2352874

**Published:** 2017-06-12

**Authors:** Jiaping Chen, Yue Jiang, Jing Zhou, Sijun Liu, Yayun Gu, Guangfu Jin, Zhibin Hu, Hongxia Ma, Hongbing Shen, Juncheng Dai

**Affiliations:** ^1^Department of Epidemiology, School of Public Health, Nanjing Medical University, Nanjing, China; ^2^Jiangsu Key Lab of Cancer Biomarkers, Prevention and Treatment, Collaborative Innovation Center for Cancer Medicine, Nanjing Medical University, Nanjing, China; ^3^State Key Laboratory of Reproductive Medicine, Nanjing Medical University, Nanjing 211166, China; ^4^Department of Social Medicine and Health Education, School of Public Health, Nanjing Medical University, Nanjing, China

## Abstract

Variants in microRNA genes may affect their expression by interfering with the microRNA maturation process and may substantially contribute to the risk of breast cancer. Recent studies have identified* miR-10b* as an interesting candidate because of its close association with the metastatic behavior of breast cancer. However, the roles of* miR-10b*-related single nucleotide polymorphisms in breast cancer susceptibility remain unclear. This case-control study evaluated the associations between variants in the upstream transcription regulation region of* miR-10b* and the risk of breast cancer among Chinese women. Seven potentially functional SNPs were investigated using genotyping assays. The potential biological functions of the identified positive SNPs were further evaluated using in silico databases. We found that rs4078756, which was located at the promoter region of* miR-10b*, was significantly associated with breast cancer risk (rs4078756 AG/GG versus AA, adjusted odds ratio: 1.17, 95% confidence interval: 1.02–1.35). The other six single nucleotide polymorphisms exhibited negative associations. Based on the in silico prediction, rs4078756 potentially regulated* miR-10b* expression through promoter activation or repression. These findings indicate that a potentially functional SNP (rs4078756) in the promoter region of* miR-10b* may contribute to breast cancer susceptibility among Chinese women.

## 1. Introduction

Breast cancer is the most common malignancy among women, with an estimated 1.7 million cases diagnosed worldwide in 2012 [1]. In China, breast cancer is the most common cancer among women and is the sixth most common cause of death among Chinese women [2]. The precise mechanisms underlying breast cancer have not been fully explored, although several strong genetic and environmental risk factors for breast cancer have been identified and have been addressed in public awareness campaigns and clinical monitoring strategies [3, 4].

Recent research has revealed that microRNAs (miRNAs) participate in human carcinogenesis as either tumor suppressors or oncogenes, and the disruption of specific miRNA expression levels and functions might play a key role in the genesis of diverse cancer types [5–7]. Abnormal expressions of many miRNAs, including* miR-34a*,* miR-210*,* miR-567*, and* miR-10b*, were also associated with breast cancer tumorigenesis or progression [8–11]. As a key molecule in the development of breast cancer,* miR-10b* was first identified as being downregulated in primary breast tumors, compared to normal breast samples [11]. However, Ma et al. reported conflicting findings in 2007, as they observed that* miR-10b* was upregulated in metastatic breast cancer [12]. Subsequent studies have revealed that overexpression of* miR-10b* triggered migration and invasion processes in various cancer cell lines, as well as distant metastasis in xenotransplantation models [12–18]. Furthermore,* miR-10b* exerted its oncogenic effects by directly targeting various tumor-associated genes, such as* HOXD10, TBX5, KLF4*, and* PTEN*, in breast cancer, pancreatic cancer, glioblastoma, and bladder cancer [12–19]. These findings indicate that* miR-10b* plays a central role in cancer metastasis and may be used as a biomarker for breast cancer carcinogenesis.

A growing number of studies have revealed that single nucleotide polymorphisms (SNPs) in miRNA genes may interfere with the miRNA transcription or maturation processes and are associated with susceptibility to cancer development [20–26]. For example, rs116159732 in the* miR-6826* primary sequence was associated with breast cancer among women of African ancestry [22]. In addition, rs11614913 in the* miR-196a2 *precursor sequence may affect the* miRNA-196a2* maturation process and is associated with the risk of breast cancer among Chinese and American women [23–25]. Furthermore, rs2682818 in the stem-loop sequence of the* miR-618* precursor may alter the secondary stem-loop structure and is associated with an increased breast cancer risk in a South American population [26]. However, we are not aware of any studies regarding the role of miR-10b SNPs in breast cancer risk. Nevertheless, given the important biological functions of miR-10b in breast cancer, polymorphisms in the* miR-10b *gene could potentially confer a risk of disease. Therefore, the present study used a case-control design to evaluate 7 potentially functional SNPs in the upstream transcription regulation region of the* miR-10b* gene. All candidate SNPs had a minor allele frequency (MAF) of ≥0.05 among Han Chinese women. We hope that the results can provide useful insights for breast cancer prevention and personalized treatment.

## 2. Materials and Methods

### 2.1. Study Population

This case-control study's protocol was approved by the Institutional Review Board of Nanjing Medical University. A total of 1,064 breast cancer cases and 1,073 cancer-free controls were included in this study, which has been described previously [27]. Briefly, the patients were recruited between January 2004 and April 2010 at the First Affiliated Hospital of Nanjing Medical University, Gulou Hospital, and Cancer Hospital of Jiangsu Province (Nanjing, China). The diagnosis of breast cancer was confirmed using pathological examination. Patients with a history of cancer, radiotherapy, or chemotherapy were excluded. Cancer-free controls were randomly selected from a pool of individuals who voluntarily participated in a community-based screening program that was performed in Jiangsu Province during the same time period. The controls had no self-reported history of cancer and were frequency-matched with the cases according to age and residential area. All subjects were genetically unrelated Han Chinese women. Approximately 95% of the eligible population provided written informed consent for participation. Each participant completed an interview using a structured questionnaire to collect information regarding the demographic characteristics, menstrual history, reproductive history, and environmental exposure history. Information regarding the estrogen receptor (ER) and progesterone receptor (PR) statuses of breast cancer cases was extracted from their medical records. After each interview, a 5 mL venous blood sample was collected from each participant.

### 2.2. SNP Selection and Genotyping Assays

The* miR-10b* gene is located at 2q31.1, which is in an intergenic region between* HOXD4* and* HOXD8* genes. Its promoter has recently been identified in human mammary cells and is located approximately 12 kb upstream of precursor* miR-10b* (pre-miR-10b) [28]. We searched the International HapMap Project (http://www.hapmap.org), dbSNP (http://www.ncbi.nlm.nih.gov/projects/SNP/), SNAP (http://archive.broadinstitute.org/mpg/snap/), and UCSC (http://genome.ucsc.edu/) databases for SNPs that were located between the promoter and pre-miR-10b. The linkage disequilibrium (LD) value (*r*^2^ < 0.8) and MAF value (≥0.05) in the Chinese Han population were also applied to select candidate SNPs. We ultimately identified nine SNPs (rs3731795, rs79025511, rs4078756, rs1348807, rs1018827, rs6736786, rs10196832, rs4972806, and rs1867863) in the upstream region of pre-miR-10b, although we omitted rs3731795 and rs79025511 because of their high LD with rs4078756 (*r*^2^ > 0.8) to optimize the assay. Thus, seven SNPs in the* miR-10b* transcript were genotyped for the present case-control study (Table 1).

The seven SNPs were genotyped using the Illumina Infinium® HumanExome BeadChip platform (Illumina, USA) and 2,137 DNA samples, which have been reported in the previous study [27]. Genotype calling was performed using Illumina's GenTrain clustering algorithm (version 1.0) in GenomeStudio (V2011.1). The genotyping call rates for all SNPs were >97% among the 1,064 breast cancer cases and the 1,073 controls. Genotyping was performed without knowledge of the individual's case or control status, and approximately equal numbers of case and control samples were tested during each assay, with two blank controls.

### 2.3. Statistical Analyses

Differences in demographic characteristics, selected variables, and genotype frequencies were compared between the cases and controls. These differences were evaluated using Student's* t-*test (equal variance assumed) for continuous variables and the *χ*^2^ test for categorical variables. The Hardy-Weinberg equilibrium was tested using the goodness-of-fit *χ*^2^ test to compare the observed and expected genotype frequencies among the control subjects.

Associations between the genotypes and breast cancer risk were estimated using logistic regression analyses adjusted for age, age at menarche, and menopausal status. The effects were reported as odds ratios (ORs) and 95% confidence intervals (CIs). All statistical analyses were performed using SAS software (version 9.1.3; SAS Institute, Cary, NC, USA). *P* values of ≤0.05 were considered statistically significant.

## 3. Results

### 3.1. Associations between the Selected SNPs and Breast Cancer Risk

The included individuals' basic characteristics are presented in Supplementary Table 1 in Supplementary Material available online at https://doi.org/10.1155/2017/2352874. After frequency matching, the cases and controls had comparable ages (*P* > 0.05). Compared to the controls, patients with breast cancer had significantly earlier menarche and later first live births (*P* < 0.0001). Among the 1,064 breast cancer cases, 490 cases (46.05%) were ER-positive and 506 cases (47.56%) were PR-positive.

The loci information and association results for the seven SNPs are described in Table 1. The multivariate logistic regression models revealed that rs4078756 was significantly associated with breast cancer risk (rs4078756 AG/GG versus AA, adjusted OR: 1.17, 95% CI: 1.02–1.35). The remaining six SNPs were not significantly associated with breast cancer risk (Table 1).

We also performed stratification analysis of the associations between rs4078756 and breast cancer risk according to age, age at menarche, age at first live birth, and menopausal status. As shown in Table 2, the breast cancer risk associated with variant AG/GG genotypes (versus the AA genotype) was significantly higher among older women (adjusted OR: 1.32; 95% CI: 1.08–1.61), postmenopausal women (adjusted OR: 1.26; 95% CI: 1.03–1.54), women with later menarche (adjusted OR: 1.29; 95% CI: 1.06–1.56), ER-positive women (adjusted OR: 1.27; 95% CI: 1.07–1.52), and PR-positive women (adjusted OR: 1.34; 95% CI: 1.12–1.60). No heterogeneity was detected for each paired comparison (*P* > 0.05).

### 3.2. Bioinformatics Analysis of the Potentially Biological Functions of rs4078756

**Figure 1:**
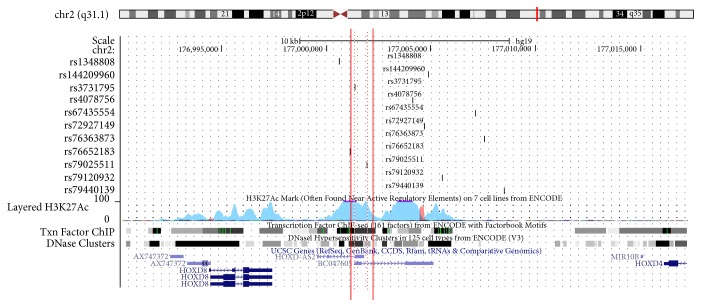
*SNPs have strong linkage disequilibrium with rs4078756 located on the promoter region of miR-10b.* The red solid line region is the promoter of miR-10b identified by Vrba et al. The rs3731795 and rs79025511 SNPs have strong linkage disequilibrium (*r*^2^ > 0.8) with rs4078756, and are located on the red dotted line region, which were strongly modified by histone H3K27Ac. These regions were enriched with transcription binding sites and DNase clusters, especially at the location of rs3731795.

The potential biological functions of rs4078756 were evaluated using bioinformatics analysis with HaploRegV4.1 and the UCSC database. As shown in Table 3, rs3731795 and rs79025511 exhibited strong linkage with rs4078756 (*r*^2^ > 0.8) in Chinese and Japanese population and were strongly modified by histone H3K27Ac, which might lead to aberrant transcription of* miR-10b* (Figure 1). Based on the JASPAR database for predicting transcription factor binding, we found that the G allele of rs3731795 might increase the binding of transcription factors, such as TCF3, TFAP2A, and TCF4, to the promoter of* miR-10b*, compared to the C allele (Table 4).

## 4. Discussion

The present study investigated the associations between breast cancer and seven potentially functional SNPs that were located in the upstream transcription regulation region of the* miR-10b* gene. The results indicate that an A-to-G base change at rs4078756 increased the risk of breast cancer among a group of Han Chinese women. To the best of our knowledge, this is the first study to evaluate the associations between breast cancer susceptibility and genetic variations in the potential regulatory region of* miR-10b*.

Previous research has indicated that* miR-10b* appears to play a key role in breast cancer invasion and metastasis. Ma et al. reported that* miR-10b* was highly expressed in clinical samples of metastatic breast cancer, and the ectopic upregulation of* miR-10b* in nonmetastatic breast cancer cells initiated invasion and metastasis [12]. Moreover,* miR-10b* silencing inhibits breast cancer metastasis in a mouse mammary tumor model [12, 13]. Additional studies have suggested that* miR-10b* regulates invasion and metastasis in breast cancer by suppressing the translation of a targeting gene* (HOXD10)* [12]. In this context,* HOXD10* is an mRNA encoding a transcriptional repressor that inhibits the expression of several genes that are involved in cell migration and extracellular matrix remodeling, such as* RhoC, uPAR, α3 integrin*, and* MT1-MMP* [12]. Furthermore,* miR-10b* could target the syndecan-1 gene and promoted breast cancer cell motility and invasiveness through a Rho-GTPase-dependent and E-cadherin-dependent mechanism [29]. Another study revealed that miR-10b promotes cell proliferation, migration, and invasion by inhibiting the expression of the* TBX5* transcription factor, which led to repression of the* DYRK1A* and* PTEN* tumor suppressor genes [19]. In addition,* miR-10b *could respond to vascular endothelial growth factor stimulation and was expressed at high levels in the human high-grade breast tumor vasculature, which suggested that vascular expression of* miR-10b* might reflect the metastatic progression of breast cancer [30].

Similar to other protein-coding genes, the* miR-10b* gene has its own promoter. The putative promoter of human* miR-10b* was initially characterized by Zhou et al., who found that it spanned between –111 bp and –460 bp upstream of pre-miR-10b [31]. Ma et al. also found that the Twist transcription factor could activate transcription of the* miR-10b* gene by binding to an E-box sequence that is proximal to its putative promoter [12]. Vrba et al. subsequently performed H3K4me3 chromatin immunoprecipitation assays using human mammary cells and redefined the promoter region of* miR-10b* as being located approximately 12 kb upstream of pre-miR-10b [28]. Several researchers have also suggested that SNPs in the promoter region of important miRNA genes may be ideal disease biomarkers. For example, our group reported that two SNPs (rs4938723 and rs999885) in the promoter region of the* miR-34b/c* and* miR-106b-25* cluster were significantly associated with the risk of hepatocellular carcinoma [32, 33]. Luo et al. also identified and confirmed an association between the rs57095329 promoter variant of* miR-146a* and systemic lupus erythematosus. Combined functional assays revealed that the risk-associated G allele of the rs57095329 had decreased binding to the Ets-1 transcription factor, which contributed to the reduced levels of* miR-146a* in patients with systemic lupus erythematosus [34]. Furthermore, the results of the present study revealed a significant association between rs4078756 in the* miR-10b* promoter region and an increased risk of breast cancer. Moreover, we found that the risk effects of the rs4078756 variant genotypes were statistically significant in some subgroups, such as women who were older, postmenopausal, ER-positive, and PR-positive and had later menarche. However, the heterogeneity tests revealed no significant heterogeneity for each paired comparison (*P* > 0.05), which suggests that these variables did not modify the risk effect.

The present study also revealed that rs4078756 is located approximately 11 kb upstream of pre-miR-10b, and is a SNP that has strong linkage with rs3731795 in the defined promoter region of miR-10b (*r*^2^ = 1). Based on the UCSC and ENCODE databases, rs3731795 lies in the maximum peak of the H3k27me3 histone mark in seven cell lines, and this mark is often found near active regulatory elements. In addition, according to JASPAR database, we observed that the G allele of rs3731795 might increase the binding of transcription factors, such as TCF3, TFAP2A, and TCF4, to the promoter of miR-10b. These transcription factors are involved in gene regulation through promoter activation or repression, depending on the specific interacting protein. However, these speculations are based on computer simulations and require confirmation using biological assays in future studies.

In conclusion, the present results suggest that rs4078756 in the promoter region of the* miR-10b *gene is associated with a significantly increased risk of breast cancer among Han Chinese women. Larger well-designed epidemiological studies with ethnically diverse populations and functional evaluations are warranted to confirm these findings.

## Supplementary Material

Socio-demographic and study variables for breast cancer cases (*n* = 1064) and controls (*n* = 1073) are presented in Supplementary Table 1. As a result of frequency matching, cases and controls were similar with respect to age. Compared with control subjects, patients with breast cancer had statistically significant earlier menarche and later first live birth (*P* < 0.0001).

## Figures and Tables

**Table 1:** Associations between seven SNPs in the miR-10b gene and breast cancer risk.

SNP	Chr	Position	Location	Alleles^a^	Cases^b^ *N* = 1,064	Controls^b^ *N* = 1,073	Call rate (%)	MAF^c^ (case/control)	HWE^d^	OR (95% CI)^e^	*P* value^e^
rs4078756	2q31.1	177,004,115	11 kb upstream of pre-mir-10b	A/G	540/436/84	582/425/66	97.80	0.285/0.260	0.341	1.17 (1.02–1.35)	0.027
rs1348807	2q31.1	177,005,757	9.3 kb upstream of pre-mir-10b	A/G	403/499/156	391/507/171	97.52	0.383/0.398	0.750	0.91 (0.80–1.03)	0.148
rs10188827	2q31.1	177,007,664	7.5 kb upstream of pre-mir-10b	A/G	514/448/99	502/471/99	97.85	0.304/0.312	0.477	0.92 (0.80–1.05)	0.210
rs6736786	2q31.1	177,008,914	6 kb upstream of pre-mir-10b	A/G	419/495/148	446/495/132	97.89	0.372/0.354	0.79	1.13 (0.99–1.29)	0.066
rs10196832	2q31.1	177,011,655	3.5 kb upstream of pre-mir-10b	A/G	936/127/1	920/143/7	97.89	0.061/0.073	0.502	0.87 (0.68–1.12)	0.281
rs4972806	2q31.1	177,012,578	2.5 kb upstream of pre-mir-10b	A/G	352/512/197	361/529/183	97.85	0.427/0.417	0.706	1.03 (0.91–1.17)	0.643
rs1867863	2q31.1	177,014,970	161 bp upstream of pre-mir-10b	A/C	434/488/140	403/519/151	97.89	0.362/0.383	0.477	0.90 (0.79–1.02)	0.105

^a^Major/minor allele; ^b^major homozygote/heterozygote/rare homozygote between cases and controls; ^c^minor allele frequency (MAF); ^d^*P*values for the Hardy-Weinberger equilibrium (HWE) test; ^e^logistic regression analysis with adjustment for age, age at menarche, and menopausal status in the additive model; Chr: chromosome, OR: odds ratio, CI: confidence interval.

**Table 2:** Associations between rs4078756 in the promoter region of miR-10b and breast cancer risk.

Characteristics	Cases			Controls			OR (95% CI)^a^	*P*	*P* ^b^
	AA (%)	AG (%)	GG (%)	AA (%)	AG (%)	GG (%)			
Age									
<51 years	312 (53.0)	242 (41.1)	35 (5.9)	289 (53.3)	226 (41.7)	27 (5.0)	1.05 (0.86–1.28)	0.648	0.111
≥51 years	228 (48.4)	194 (41.2)	49 (10.4)	293 (55.2)	199 (37.5)	39 (7.3)	1.32 (1.08–1.61)	0.007	
Menopausal status									
Premenopausal	266 (51.9)	211 (41.1)	36 (7.0)	267 (53.0)	212 (42.1)	25 (5.0)	1.15 (0.93–1.42)	0.197	0.540
Postmenopausal	217 (48.2)	194 (43.1)	39 (8.7)	286 (54.5)	201 (38.3)	38 (7.2)	1.26 (1.03–1.54)	0.028	
Age at menarche									
<16 years	320 (53.5)	236 (39.5)	42 (7.0)	217 (52.8)	172 (41.9)	22 (5.4)	1.03 (0.84–1.26)	0.799	0.115
≥16 years	211 (47.5)	192 (43.2)	41 (9.2)	363 (55.0)	253 (38.3)	44 (6.7)	1.29 (1.06–1.56)	0.010	
Age at first live birth									
<24 years	119 (49.6)	104 (43.3)	17 (7.1)	204 (55.0)	140 (37.7)	27 (7.3)	1.18 (0.90–1.53)	0.229	0.831
≥24 years	392 (52.0)	301 (39.9)	61 (8.1)	358 (53.4)	276 (41.1)	37 (5.5)	1.14 (0.96–1.36)	0.141	
ER status									
Positive	237 (48.6)	211 (43.2)	40 (8.2)				1.27 (1.07–1.52)	0.008	0.713
Negative	194 (51.5)	146 (38.7)	37 (9.8)				1.21 (1.0–1.46)	0.055	
PR status									
Positive	237 (46.9)	223 (44.2)	45 (8.9)				1.34 (1.12–1.60)	0.001	0.204
Negative	192 (53.3)	136 (37.8)	32 (8.9)				1.13 (0.93–1.37)	0.235	

^a^Per-allele odds ratio (OR) and 95% confidence interval (CI) adjusted for age, age at menarche, and menopausal status where appropriate; ^b^*P* value for the heterogeneity test; ER: estrogen receptor; PR: progesterone receptor.

**Table 3:** Annotation of variants with strong linkage disequilibrium with the SNP rs4078756 in HaploRegV4.1.

Chr	Pos (hg19)	LD (*r*^2^)	Variant	Ref	Alt	ASN freq	Promoter histone marks	DNAse	Proteins bound	Motifs changed
2	177000616	1.00	rs1348808	T	C	0.23	15 tissues	12 tissues	CTBP2	Ets, LF-A1, NF-E2
2	177001145	1.00	rs76652183	G	T	0.23	19 tissues	18 tissues	5 bound proteins	EBF
2	177001378	1.00	rs3731795	A	G	0.27	19 tissues	7 tissues		CTCF, p300
2	177001962	0.96	rs79025511	C	T	0.23	19 tissues	MUS, MUS		Gcm1
2	177004689	0.92	rs72927149	G	C	0.23	12 tissues	6 tissues		Pax-5
2	177005519	1.00	rs79120932	G	T	0.23	19 tissues	32 tissues	5 bound proteins	4 altered motifs
2	177007102	1.00	rs67435554	G	T	0.23				
2	177007527	1.00	rs76363873	A	G	0.23	IPSC			GATA, MZF1::1–4
2	177008484	1.00	rs79440139	T	A	0.23				6 altered motifs

Chr: chromosome, Pos: position, LD: linkage disequilibrium in CHB + JPT population, Ref: reference, Alt: alternative, freq: frequency, MUS: musculus, and IPSC: induced pluripotent stem cell.

**Table 4:** The SNP rs3731795 might influence the binding of transcription factors in JASPAR database.

Motif	Relative score		Start	End	Strand	Predicted
	A allele	G allele				site sequence
TCF3	0.610	0.804	11	20	−1	GTGACTG
TFAP2A	0.685	0.873	11	19	1	CGGTGA
TCF4	0.649	0.807	11	20	−1	TCCAGTCACC
NRF1	0.648	0.805	8	18	−1	AGCCGGTGA
TFAP2A	0.767	0.875	8	16	−1	AGCCGGTGACT
TFAP2A	0.757	0.801	10	20	−1	TGACTGG
