# Supplementary material for: Genetic Variants in the Promoter Region of miR-10b and the Risk of Breast Cancer
Source: Biomed Res Int. 2017 Jun 12;2017:2352874. doi: 10.1155/2017/2352874 (PMC5485289; doi:10.1155/2017/2352874)
Supplement: Supplementary file 1 — Socio-demographic and study variables for breast cancer cases (n = 1064) and controls (n = 1073) are presented in Supplementary Table 1. As a result of frequency matching, cases and controls were similar with respect to age. Compared with control subjects, patients with breast cancer had statistically significant earlier menarche and later first live birth (P < 0.0001). [file 2352874.f1.doc]

**Supplementary Table 1. Demographic and selected variables in breast cancer and control patients**

| **Variables** | **Patients^a^** | **Controls^b^** | ***P^c^*** |
| --- | --- | --- | --- |
|  | **(N=1064)** | **(N=1073)** |  |
| Age, year (mean ± SD) | 50.89±11.44 | 51.51±11.82 | 0.217 |
| Age at menarche, year (mean ± SD) | 15.23±1.93 | 16.11±1.94 | <0.0001 |
| Age at ﬁrst live birth, year (mean ± SD) | 25.57±3.25 | 24.62±3.35 | <0.0001 |
| Age at natural menopause, year (mean ± SD) | 49.72±3.53 | 49.59±3.98 | 0.606 |
| Menopausal status |  |  | <0.0001 |
| Premenopausal | 523 | 505 |  |
| Natural menopause | 451 | 525 |  |
| Unnatural menopause | 78 | 23 |  |
| Estrogen receptor (ER)^d^ |  |  |  |
| Positive | 490 |  |  |
| Negative | 379 |  |  |
| Progesterone receptor (PR) ^d^ |  |  |  |
| Positive | 506 |  |  |
| Negative | 363 |  |  |

^a^ Patients were consecutively recruited from the First Affiliated Hospital of Nanjing Medical University, the Cancer Hospital of Jiangsu Province and the Gulou Hospital, Nanjing, China, from Jan 2004 to April 2010;

^b^ Controls were randomly selected from a cohort of more than 30,000 participants in a community-based screening program for non-infectious diseases conducted in Jiangsu Province;

^c^ T-tests and *χ*2 tests were used for continuous or categorical variables, respectively;

^d^ ER and PR status information was available in 869 breast cancer cases.
